# Predicting infectious etiology and severity in hospitalized pediatric pneumonia using blood cytokine biomarkers

**DOI:** 10.3389/fped.2025.1693879

**Published:** 2025-12-19

**Authors:** Alexis M. Duray, Benjamin Lee, Robert N. Abood, Samar Musa, Sophia Kainaroi, Marian G. Michaels, Jason E. Shoemaker, John F. Alcorn

**Affiliations:** 1 Division of Pulmonary Medicine, Department of Pediatrics, UPMC Children’s Hospital of Pittsburgh, Pittsburgh, PA, United States; 2 Division of Infectious Diseases, Department of Pediatrics, UPMC Children’s Hospital of Pittsburgh, Pittsburgh, PA, United States; 3 Department of Chemical and Petroleum Engineering, University of Pittsburgh, Pittsburgh, PA, United States

**Keywords:** virus, bacteria, influenza, RSV (respiratory syncytial virus), rhinovirus (RV)

## Abstract

**Background:**

Lower respiratory infections are a significant cause of morbidity and mortality in children. The aim of this study was to determine whether cytokine levels measured in plasma at the time of admission to the hospital can predict disease etiology or severity.

**Methods:**

Blood was collected from pediatric inpatients, and cytokine levels were determined by cytokine multiplex analyses. Plasma cytokine concentrations were then analyzed using logistic regression and machine learning approaches to determine if we could accurately predict if a child would require longer-term hospitalization (≥5 days), intensive care, or exhibit hypoxemia (SpO_2_ < 90%).

**Results:**

A total of 159 patients were enrolled, and 59 cytokines were assessed in relation to the type of infection and severity. The most prevalent viral infections were human rhinovirus/enterovirus (hRV/EV; 24.4%), respiratory syncytial virus (RSV; 21.8%), and influenza virus (16.7%). Several cytokines (CHI3L1, IL-1Rα, IL-6, G-CSF, MCP-1, and MIP-1α) were elevated in severe pneumonia cases, regardless of disease etiology. Predictors of duration in RSV cases were distinct from other causes, with a predominance of type-2 immune response. Cytokines such as chitinase-3-like-1 (CHI3L1), pentraxin-3, osteopontin, and IL-20 correlated with severity across multiple groups. Plasma levels of IL-6, MMP-2 and LIGHT could be employed to separate viral vs. community acquired pneumonia (CAP). In influenza cases, longer-term hospitalization and ICU admission could be predicted based on two cytokines, CHI3L1 and sTNFR1. RSV severity was closely correlated with levels of MIP-1α, IL-26, G-CSF, and IFNβ.

**Conclusions:**

This study highlights the heterogeneity of immune responses to severe pneumonia and provides new groupings of cytokines which may distinguish between viral and non-viral pneumonia.

## Introduction

Respiratory infections are among the most frequent health problems during childhood. On average, a child will experience 22 respiratory infections within the first 12 years of life (1). Lower respiratory infections account for the greatest negative impact in disability-adjusted life years (DALYs) in children under the age of 10 (2). Infection severity can vary greatly among those infected, with some going on to develop pneumonia and requiring hospitalization. In the United States, the incidence of hospitalization for pneumonia varies yearly, with some estimating approximately 16 hospitalizations per 10,000 children (3).

Although vaccination against pneumococcus and *Haemophilus influenzae* type B has reduced the burden of bacterial community-acquired pneumonias (CAPs), the contribution of viral-associated pneumonias is increasing (4). Viral pathogens can be detected in 55%–66% of pediatric pneumonia cases (3, 4). The most frequently detected viruses are respiratory syncytial virus (RSV), human rhinovirus/enterovirus (hRV), human metapneumovirus (hMPV), and influenza virus (Flu). Many studies have noted the impact of parainfluenza viruses, coronaviruses, and adenoviruses, although these viruses have not been consistently evaluated (3–6).

The ability to predict disease severity for any given pneumonia at the time of recognition, regardless of etiology, would be an invaluable clinical tool. Current standards upon admission include testing for C-reactive protein (CRP) and procalcitonin levels. While CRP is exceedingly sensitive in detecting inflammation, it lacks specificity for infection and can reflect other inflammatory conditions (7, 8). High CRP levels may offer more reliability in predicting bacterial infections, particularly sepsis, but are not good indicators of viral infections (8). Procalcitonin levels are good indicators of bacterial infection and viral–bacterial coinfection, but are poor indicators of viral pneumonia (9–11). Inflammatory cytokines have been associated with viral pneumonia with variable predictive value (12–14). Thus, there remains a gap in the field to reliably predict viral pneumonia and severity.

In this study, we analyzed blood samples from pediatric patients hospitalized for pneumonia to determine the etiology or predict severity of infection. We isolated plasma from patients within 48 h of hospitalization. We then assessed cytokine levels in the plasma and compared them to known infectious agents and various metrics of disease severity [duration of hospitalization, admittance to the intensive care unit (ICU), and blood oxygenation (SpO_2_) levels].

## Methods

### Human participants and eligibility

For this study, participants were screened for eligibility at the UPMC Children's Hospital of Pittsburgh based on a defined set of inclusion and exclusion criteria. Inclusion criteria were as follows: (1) age >28 days; (2) weight >3 kg; (3) hospitalization; (4) clinical diagnosis of acute respiratory infection, defined as fever (temperature ≥38°C/100.4°F) within ± 24 h of admission with subjective symptoms and/or objective signs localizable to the upper or lower respiratory tract in the absence of alternate explanation; and (5) a planned clinical blood draw for medical management or existing intravenous catheter through which blood could be drawn if no additional blood draws were planned. Once all inclusion criteria were met, potential participants were excluded if they had one or more of the following: (1) known or suspected primary immunodeficiency; (2) secondary or acquired immunodeficiency or immunocompromised status, including chronic steroid therapy (>2 mg/kg/day or >20 mg/day if weight >10 kg of systemic, i.e., oral or intravenous, steroid therapy for >14 days), active or recent chemotherapy (within 90 days), active or recent treatment with immune-modifying or immunosuppressive agents (within 90 days), history of hematopoietic stem cell transplantation, history of solid organ transplantation, or known infection with human immunodeficiency virus.

Participants were enrolled between October 2013 and December 2019 and again between October 2023 and April 2024. Enrollment was paused during the COVID-19 pandemic to attempt to exclude SARS-CoV-2-positive cases. If patients had a known test positive for SARS-CoV-2 infection, they were not enrolled. Standardized medical chart reviews were conducted for each participant to collect demographic data and additional clinical findings. This study was conducted in accordance with the principles of the Declaration of Helsinki and was approved by the University of Pittsburgh Institutional Review Board (CR19090225). All participants provided their written informed consent before enrollment.

### Infectious agent identification

Participants were screened for common respiratory viruses using either a clinical standard-of-care respiratory viral panel or real-time quantitative reverse-transcription polymerase chain reaction (RT-qPCR) assays utilizing primers, probes, and testing protocols developed by the Centers for Disease Control and Prevention (CDC) in collaboration with the New Vaccine Surveillance Network (NVSN). Bacterial culture data were obtained from clinical samples only.

### Whole blood processing

Whole blood was collected within 48 h of admission. Blood samples were processed using mononuclear cell preparation tubes (CPT; BD Biosciences, Franklin Lakes, NJ, USA) to collect both cells and plasma. Plasma was aliquoted and frozen at −80°C.

### Plasma cytokine analysis

Cytokine levels in plasma were measured using the Bio-Plex Pro Human Inflammation Panel 1, 37-plex, and the Bio-Plex Pro Human Cytokine 27-plex Assay (Bio-Rad, Hercules, CA, USA) according to the manufacturer's instructions. The protein targets measured across the assays included the following: APRIL (TNFSF13), BAFF (TNFSF13B), sCD30 (TNFRSF8), sCD163, chitinase-3-like 1 (CHI3L1), gp130 (sIL-6Rβ), IFN-α2, IFN-β, IFN-γ, IL-1β, IL-1ra, IL-2, IL-4, IL-5, IL-6, sIL-6Rα, IL-7, IL-8, IL-9, IL-10, IL-11, IL-12 (p40), IL-12 (p70), IL-13, IL-15, IL-17, IL-19, IL-20, IL-22, IL-26, IL-27 (p28), IL-28A (IFN-λ2), IL-29 (IFN-λ1), IL-32, IL-34, IL-35, LIGHT (TNFSF14), MMP-1, MMP-2, MMP-3, osteocalcin, osteopontin, pentraxin-3, sTNF-R1, sTNF-R2, TSLP, TWEAK (TNFSF12), FGF basic, eotaxin, G-CSF, GM-CSF, IP-10, MCP-1, MIP-1α, MIP-1β, PDGF-BB, RANTES, TNF-α, and VEGF. For cytokines represented in both panels, only values from the 37-plex assay were reported. Cytokine concentrations were natural log-transformed throughout the figures.

### Statistical and machine learning analysis

Differences in demographic data, duration of hospitalization, ICU admission, and hypoxemic status within the entire cohort, or within specific infections, were assessed using Fisher's exact test. Cytokine levels were correlated with duration of hospitalization and SpO_2_ levels to identify candidates. All significantly correlated cytokines were then subjected to multiple linear regressions against age and sex. Any cytokines which correlated with age or sex (*p* ≤ 0.05) were excluded from downstream analysis. For categorical analysis of cytokine levels (longer- vs. shorter-term stays: ±5 days, ICU admission: yes/no, hypoxemia: yes/no), Welch's *t*-tests were performed to identify differences. Any significant data from those tests were then subjected to more stringent statistical analysis, including false discovery rate (FDR) correction (significance denoted as *q*-value), as well as linear and logistic regressions, to identify candidates. Multiple logistic regression was performed on cytokine groups (1–4 cytokines, with and without two-way interactions) to generate receiver operating characteristic (ROC) curves. Heatmaps represent mean values. Graphs represent mean ± SEM. Statistical analysis, including principal component analysis (PCA), was conducted using GraphPad PRISM software (San Diego, CA, USA).

Machine learning analyses were conducted using a Random Forest classifier implemented in Python. The RandomForestClassifier was utilized and cross-validation (*k* = 3) was performed using KFold and cross_val_score. All algorithms were sourced from the scikit-learn library. The model was trained on the input dataset to predict the target variable. Hyperparameters were maintained at default levels, except for the maximum depth, which was set to 5. Feature importance was performed by iterative exploration. The area under the curve of the ROC (ROC AUC) was used as the performance metric.

## Results

### Participant cohort characteristics

Participants were enrolled based on eligibility criteria defined previously. A total of 159 participants were enrolled in this study. After enrollment, two participants chose to withdraw, and one was identified as a readmission and excluded from analysis. Demographic data are provided in Table 1. Among the enrolled participants, children with RSV were significantly younger than any other cohort. Overall, 59.6% of the cohort required ICU admission with significantly different infection etiologies; the highest were observed in hRV/EV and RSV cases.

**Table 1:** ARI study cohort characteristics.

Feature	Overall (*n* = 156)	hRV/EV (*n* = 38)	RSV (*n* = 34)	Influenza (*n* = 26)	hMPV (*n* = 8)	Other/undetermined (*n* = 17)	CAP (*n* = 33)	*p*-Value
Sex (%)								
Female	48.1	57.9	50.0	61.5	37.5	58.8	42.4	0.6011
Male	51.9	42.1	50.0	38.5	62.5	41.2	57.6	
Age (years)								
Average	5.35	5.99	1.55	6.85	4.33	3.95	8.29	<0.0001
Median (range)	4.18 (0.1–24.9)	5.55 (0.22–12.56)	0.96 (0.16–6.05)	6.10 (0.17–24.99)	2.34 (1.36–17.5)	1.36 (0.50–15.75)	8.55 (0.1–16.2)	
Race (%)								
White	80.8	76.3	70.6	92.3	87.5	70.6	90.9	0.1157^a^
Black	13.5	21.1	14.7	7.7	12.5	17.6	9.1	
Other/declined	5.8	2.6	14.7	0	0	11.8	0	
Ethnicity (%)								
Hispanic	3.2	0	2.9	0	12.5	11.8	3	0.0743^a^
Non-Hispanic	84	89.5	64.7	100	87.5	82.4	87.9	
Declined	12.8	10.5	32.4	0	0	5.9	9.1	
Hospital duration (days)								
Median (range)	3 (0–67.9)	3.5 (0.9–25.9)	4 (0.7–24)	3 (1–67.9)	4 (1–38.1)	2 (0–11)	3 (0.8–29.2)	0.1610
ICU admission (%)								
Yes	59.6	81.6	73.5	42.3	62.5	35.3	45.5	0.0007
No	40.4	18.4	26.5	57.7	37.5	64.7	54.5	
Hypoxemic (SpO_2_ < 90%), %								
Yes	40.4	44.7	52.9	38.5	50.0	35.3	24.2	0.2427^a^
No	59.0	55.3	47.1	57.7	50.0	64.7	75.8	
Untested	0.6	0.0	0.0	3.8	0.0	0.0	0.0	
Sample collected (%)								
Yes	73.7	73.7	73.5	84.6	75.0	41.2	78.8	
No	26.3	26.3	26.5	15.4	25.0	58.8	21.2	

a Other/declined/untested were excluded from comparison.

### Cytokines associated with severity in all pneumonia cases

**Figure 1:**
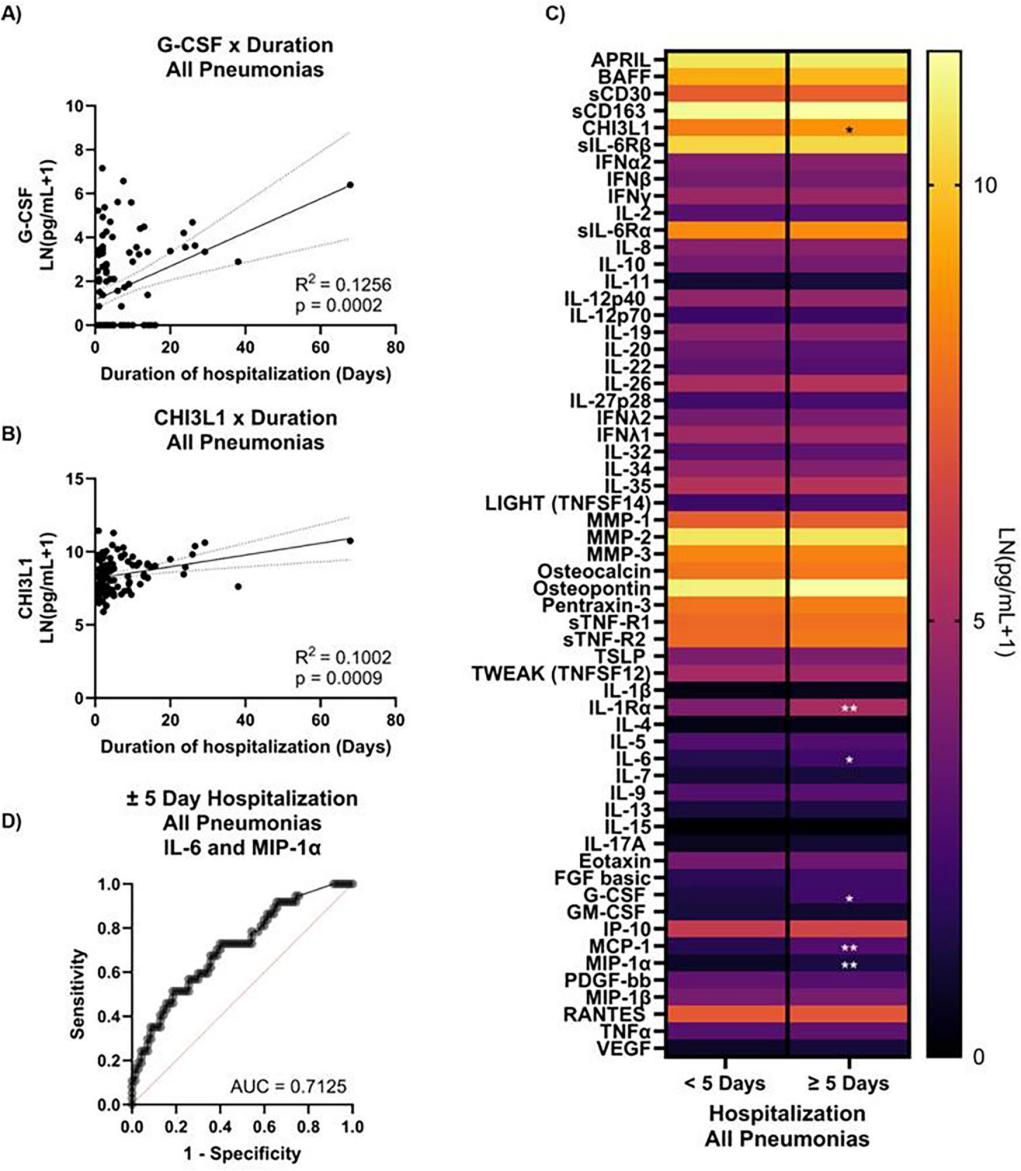
Biomarkers for duration of hospitalization in pneumonia inpatients. Plasma levels of **(A)** G-CSF and **(B)** CHI3L1 were plotted against participant duration of hospitalization (days) and linear regression performed to determine correlation. **(C)** Plasma cytokines were also compared between participants with shorter- (<5 days) vs. longer-term (≥5 days) hospitalization. **(D)** ROC curve generated by logistic regression using IL-6 and MIP-1α and IL-6:MIP-1α levels to classify shorter- vs. longer-term hospitalization (*n* = 107). *q < 0.05, **q < 0.01.

We analyzed the plasma cytokines levels of 115 participants for which a blood sample was obtained to determine if any cytokine(s) tightly correlated with the duration of hospitalization, admission to the ICU, or hypoxemia (SpO_2_ < 90%) as proxies for disease severity. Eleven cytokines significantly linearly correlated with duration of hospitalization, of which only five had an *R*^2^ > 0.1. Three of these cytokines—IL-20, IL-1Rα, and MCP-1—also correlated with age, leaving only CHI3L1 and G-CSF as predictors of duration (Figures 1A,B), though they are relatively weak considering the low *R*^2^ values. We also assessed changes in cytokine levels between longer- and shorter-term hospitalization, using the median stay (3 days) and a more severe cutoff (5 days). For ±3-day hospitalization, eight cytokines trended toward being different (Supplementary Figure S1). When the more stringent ±5-day hospitalization was used, six cytokines were significantly different (MIP-1α, IL-1Rα, MCP-1, IL-6, G-CSF, and CHI3L1; Figure 1C and Supplementary Figure S1). As we previously observed IL-1Rα and MCP-1 levels to correlate with age, they were excluded from further analysis. Using IL-6 and MIP-1α, we could correctly classify longer- or shorter- term stays (±5-day) 72% of the time (Figure 1D).

**Figure 2:**
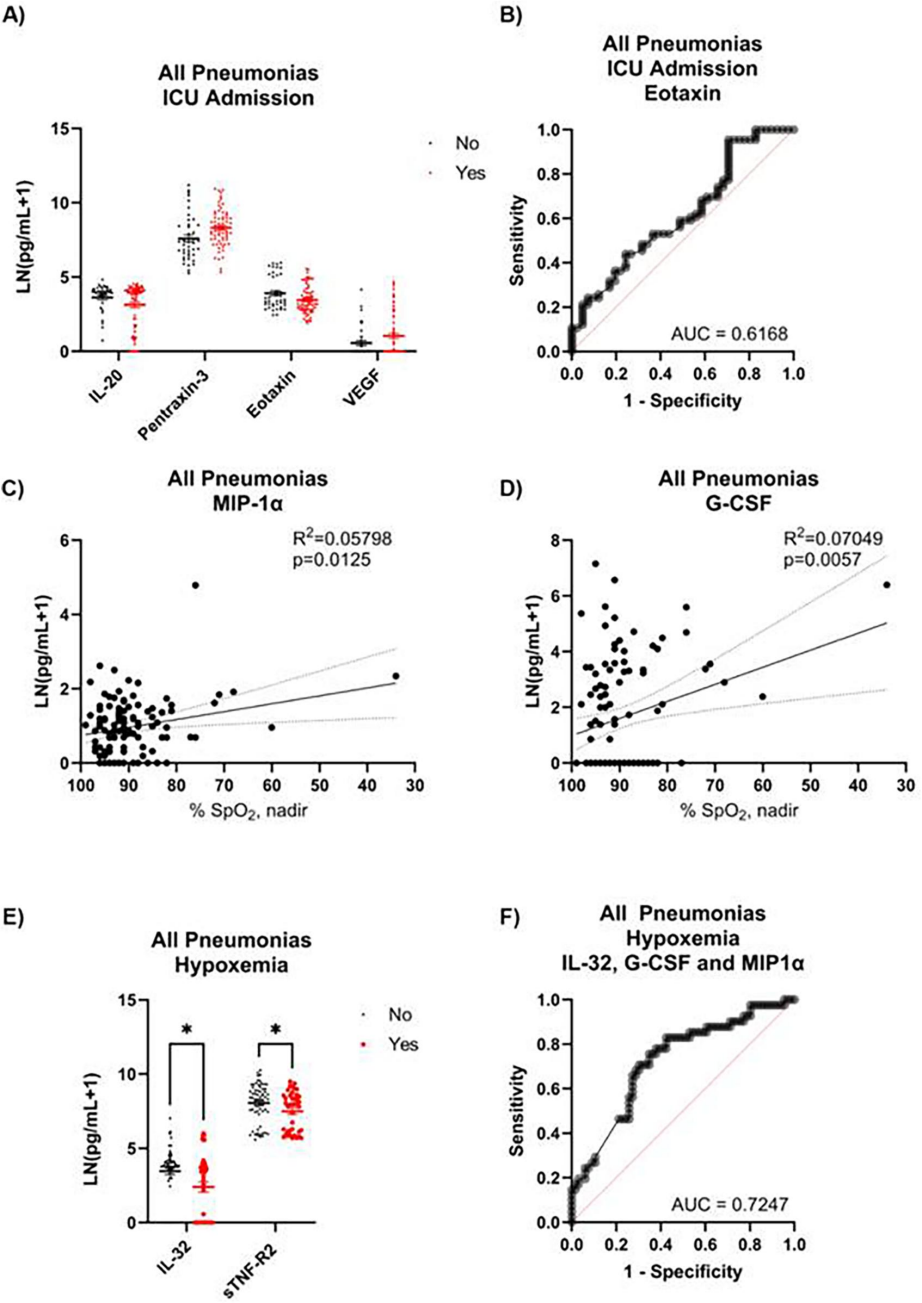
Biomarkers that track with ICU admission and hypoxemia in pneumonia inpatients. **(A)** Participants were separated by ICU admission and plasma cytokines measured. **(B)** ROC curve generated by logistic regression for eotaxin against ICU admission. Blood oxygenation (% SpO_2_) was correlated against **(C)** MIP-1α and **(D)** G-CSF via linear regression. **(E)** Cytokines were also measured against hypoxemic status (SpO_2_ ± 90%). **(F)** ROC curve generated by logistic regression for IL-32, G-CSF, and MIP-1α, plus two-way interactions, against hypoxemic status (*n* = 107). *q < 0.05.

For those admitted to the ICU, four cytokines tended to be different, with two elevated and two decreased (Figure 2A). After accounting for age and sex, VEGF no longer correlated with ICU admission (*p* = 0.0652), and IL-20 and pentraxin-3 correlated with age. Eotaxin alone was modestly successful at predicting ICU admission, with 63.6% of cases correctly identified (Figure 2B). G-CSF and MIP-1α correlated with SpO_2_ levels, though the associations were weak (Figures 2C,D). Individuals with hypoxemia had lower levels of IL-32 and sTNF-R2 (Figure 2E). IL-32 coupled with G-CSF and MIP-1α was able to correctly classify hypoxemic status 66.4% of the time (Figure 2F). Given the highly heterogeneous nature of the infectious agents, we next sought to assess if these cytokines might have greater predictive power once analyzed within each infection type.

### Differentiation of viral vs. non-viral pneumonia via plasma cytokines

**Figure 3:**
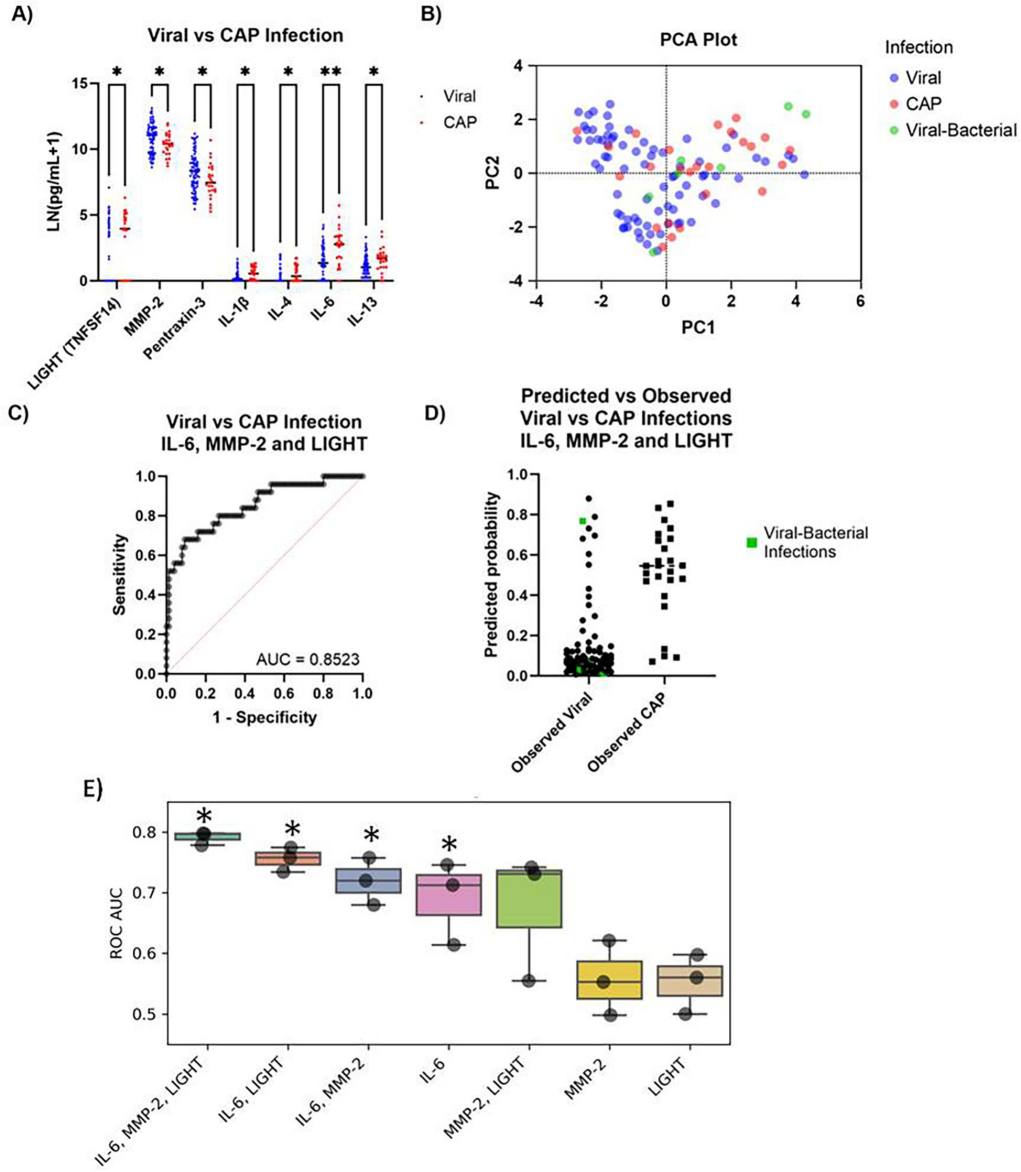
Differentiation of viral vs. CAP patients using cytokine biomarkers. **(A)** Cytokines significantly different between confirmed viral vs. CAP infections. **(B)** PCA for infection and levels of cytokines identified in **(A)** (PC1 – 48.15%, PC2 – 13.9%). **(C)** ROC curve generated by logistic regression classifying viral or CAP infection based on IL-6, MMP-2, and LIGHT levels, plus two-way interactions. **(D)** Predicted probability of infection based on logistic regression in **(C)**, with confirmed viral–bacterial infections highlighted in green (*n* = 99, 107). **(E)** Boxplot of the receiver operator curve area under the curve (ROC AUC) when using a Random Forrest (RF) model to predict viral vs. non-viral infection. For each protein or protein combination shown, threefold cross-validation was performed. *q < 0.05 **q < 0.01.

For decades, extensive efforts have focused on finding simple biomarkers which can differentiate between viral and non-viral pneumonias for quick diagnosis and execution of appropriate treatment protocols. In our small cohort, we assessed cytokine levels between confirmed viral infections and CAP. Seven participants had confirmed viral and bacterial infections and were excluded from initial analysis. A total of seven cytokines had differential expression between virus and CAP cases (Figure 3A). We then performed principal component analysis on these seven cytokines and determined infection status (Figure 3B). Using levels of IL-6, LIGHT, and MMP-2, we differentiated viral infections correctly 95% of the time and CAP infection 54% of the time, yielding an overall accuracy of classification of 83%, a positive predictive power of 81.25%, and a negative predictive power of 83.33% (Figure 3C). When the coinfected individuals were incorporated into this model, all but one separated to predicted viral infection (Figure 3D). Finally, threefold cross-validation was performed using a Random Forrest (RF) algorithm, with all combinations of IL-6, LIGHT, and MMP-2, to determine which combinations could most accurately predict whether a patient had viral or non-viral pneumonia (Figure 3E). An RF model trained to IL-6 data alone was significantly more accurate in predicting the case type than using MMP-2 or LIGHT alone. RF models trained to all combinations including IL-6 data demonstrated superior performance.

### Viral pneumonia etiology by plasma cytokines

**Figure 4:**
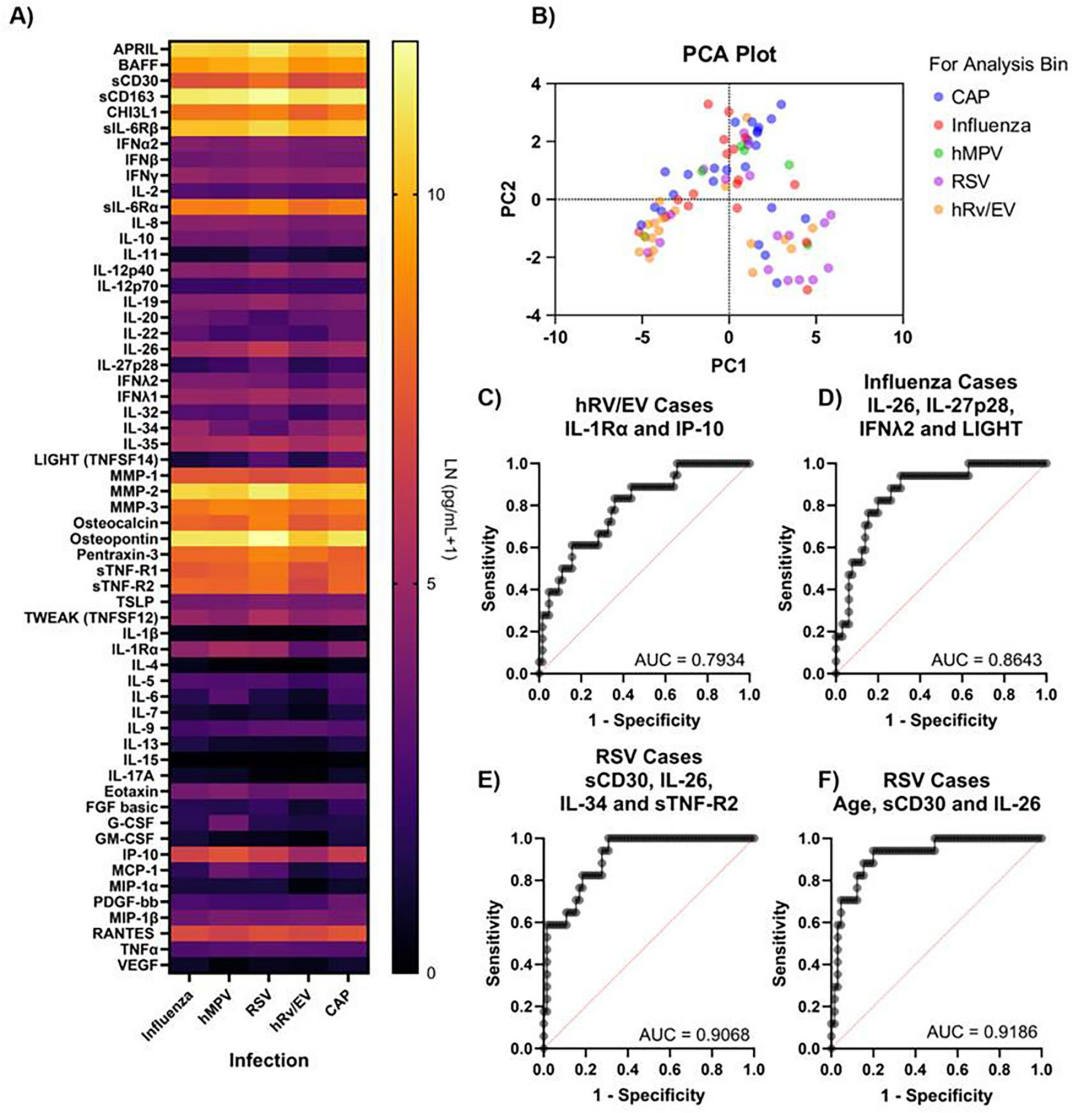
Plasma cytokine predictors of viral pneumonia etiology. **(A)** Plasma cytokine levels compared against specific infection identified. **(B)** PCA analysis of significantly different cytokines against infection. ROC curves generated by logistic regressions classifying specific infection for **(C)** hRV/EV cases, using IL-1Rα, IP-10, and IL-1Rα:IP-10, **(D)** Influenza cases, using IL-26, IL27p28, IFNλ2, LIGHT, and two-way interactions and RSV cases using **(E)** sCD30, IL-26, IL-34, and sTNF-R2 levels, or **(F)** age (years), sCD30, IL-26, and sCD30:IL-26.

We then assessed our ability to differentiate between specific viral and CAP infections in our cohort. We compared cytokine profiles of confirmed viral infections and CAP infections for which there was no coinfection identified (viral–viral, viral–bacterial, or bacterial–bacterial) (Figure 4A). A total of 21 cytokines showed a significant difference between the various infections (Supplementary Table S1). These 21 cytokines were subjected to principal component analysis to differentiate the various types of infections (Figure 4B). We did not observe clearly defined clusters based on these cytokines across the different infections, though hRV/EV infections tended to cluster in the lower left quadrant and RSV in the lower right quadrant, with influenza, CAP, and hMPV cases scattered throughout. We performed multiple logistic regressions against the cytokines identified in Supplementary Table S1 to determine if these cytokines would be good predictors of infectious etiology. We noted that IL-1Rα and IP-10 represented the best cytokine pair for differentiating hRV/EV cases from other infections (Figure 4C). Although this test correctly identified 95% of negative cases, it only predicted 27.8% of positive cases, indicating that the test lacks specificity for hRV/EV. Prediction of influenza cases was moderately better, but required more cytokines, including IL-26, Il-27p28, IFNλ2, and LIGHT (Figure 4D). Removal of any of these cytokines resulted in a dramatic decrease in model prediction of positive cases—from 47% to as low as 11%. RSV case prediction was the most successful, with 59% of infected individuals correctly identified. Overall, 90% of all cases were correctly identified using sCD30, IL-26, IL-34, and sTNF-R2 (Figure 4E). Consistent with established findings in the field and our dataset, RSV infection was most prevalent in very young children. When age was included as a predictor in our model, coupled with sCD30 and IL-26, we achieved the same predictive power as with the combination of the abovementioned four cytokines (Figure 4F). Given the low number of hMPV cases in our dataset, we did not attempt to differentiate hMPV cases.

### Cytokines associated with severity in hRV/EV cases

**Figure 5:**
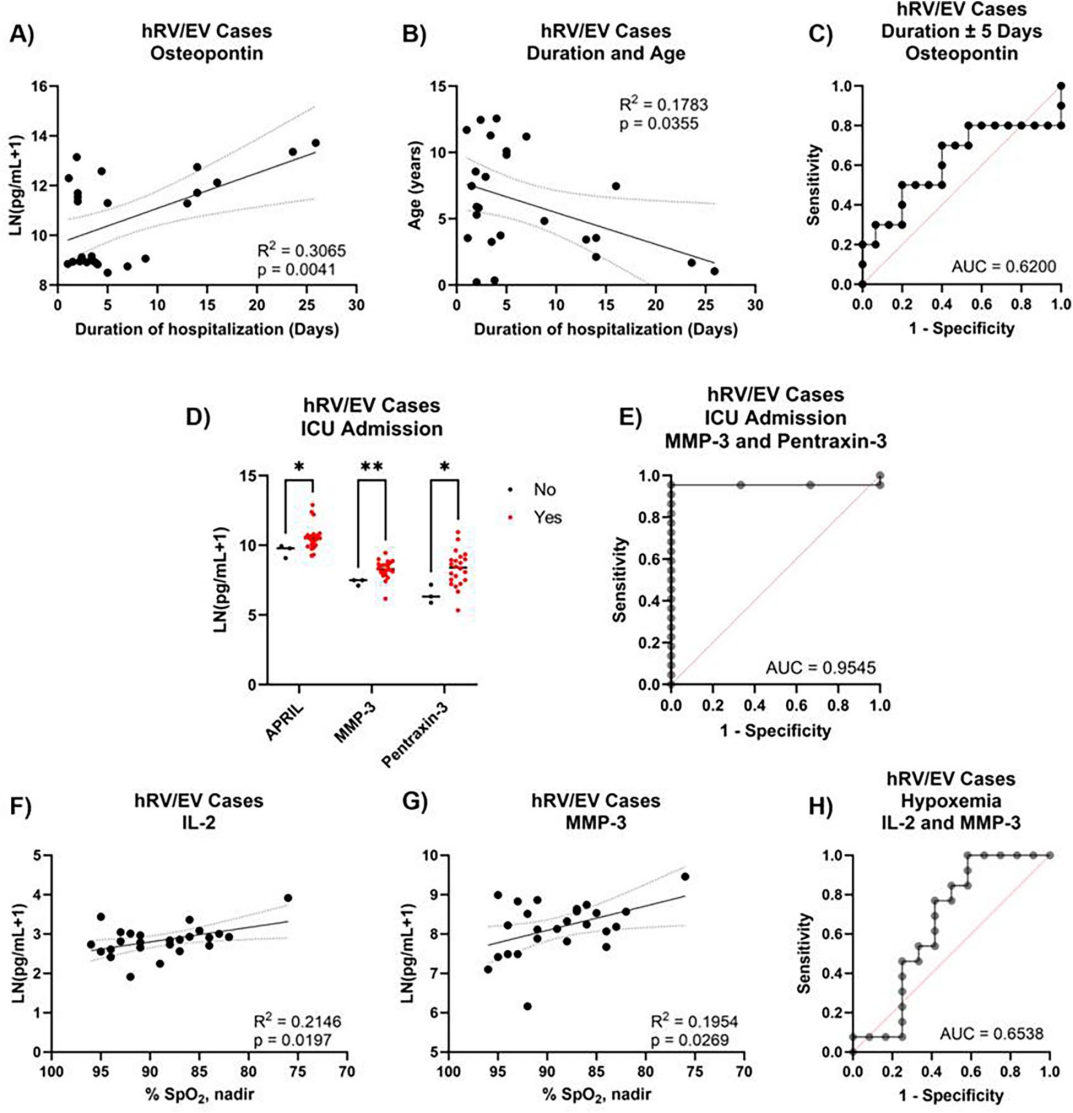
Plasma cytokine predictors of hRV/EV severity. Linear regression of **(A)** Osteopontin (OPN) levels and **(B)** age against duration of hospitalization. **(C)** ROC curve generated by logistic regression using OPN levels to classify ±5-day hospital duration. **(D)** Cytokine levels between participants with confirmed hRV/EV cases against ICU admission. **(E)** ROC curve generated by logistic regression using MMP-3, pentraxin-3, and MMP-3:pentraxin-3 to classify ICU admission. Linear regression of **(F)** IL-2 and **(G)** MMP-3 levels against % SpO_2_ levels. **(H)** ROC curve generated by logistic regression using IL-2 and MMP-3 to classify hypoxemic status (*n* = 25). *q < 0.05 **q < 0.01.

Many of the participants in our cohort had a confirmed hRV/EV infection. Possibly due to the large size of the cohort, we found 22 cytokines that correlated with duration of hospitalization, 14 of which had an *R*^2^ > 0.2 (Supplementary Figure S2). After accounting for age and sex, only osteopontin (OPN) remained significantly correlated with duration (Figure 5A). This is likely attributable to the significant correlation between duration of hospitalization and age in our cohort (Figure 5B). OPN was only modestly successful at predicting longer- vs. shorter-term hospitalization on its own, with 64% of cases correctly classified (Figure 5C). Assessment of ICU predictors was also challenging due to the limited number of non-admitted cases (3/25). Five cytokines were different between those admitted to the ICU and those not, of which APRIL, MMP-3, and pentraxin-3 remained correlated when accounting for age and sex (Figure 5D). Using MMP-3 and pentraxin-3, 92% of cases were correctly classified on ICU admission (Figure 5E). The incidence of hypoxemia was more equitably distributed across our cohort of participants, with roughly half of the cases having SpO_2_ of <90% during hospitalization (12/25). Levels of APRIL, IL-2, and MMP-3 correlated with SpO_2_ levels, though the association with APRIL was lost after accounting for age and sex (Figures 5F,G). IL-2 and MMP-3 were poor predictors of hypoxemia (Figure 5H).

### Cytokines associated with RSV severity

**Figure 6:**
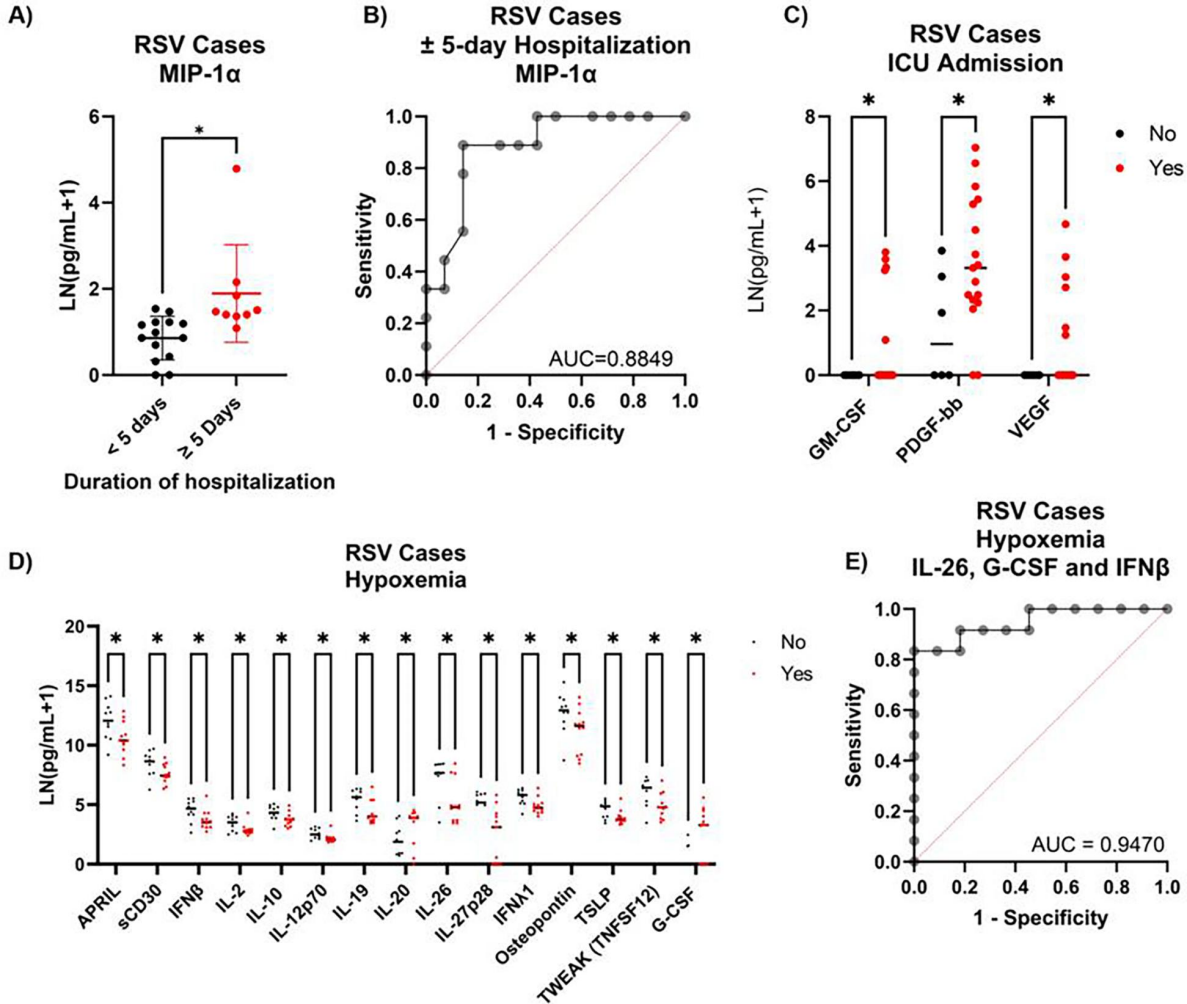
Plasma cytokine predictors of RSV severity. **(A)** Levels of plasma MIP-1α in RSV^+^ cases between those hospitalized for ±5 days. **(B)** ROC curve generated by logistic regression using MIP-1α levels alone to classify RSV^+^ cases with shorter- vs. longer-term hospitalization times. Cytokines significantly different between RSV^+^ cases **(C)** admitted to the ICU or not and **(D)** with or without hypoxemia (SpO_2_ ± 90%). **(E)** ROC curve generated by logistic regression classifying hypoxemic status in RSV^+^ cases using IL-26, G-CSF, IFNβ, and 2-way interactions (*n* = 23).

Within only RSV^+^ individuals (*N* = 34), we found nine cytokines that significantly correlated with duration of hospitalization, six of which remained correlated after controlling for age and sex (Supplementary Figure S3). Another cytokine, MIP-1α, was significantly higher in those hospitalized for ≥5 days and was also included in downstream analysis (Figure 6A). MIP-1α levels alone could reliably predict longer- vs. shorter-term stays in nearly 87% of cases (Figure 6B). None of the other cytokines that correlated with duration surpassed this model alone, nor enhanced it when combined. MIP-1α was not, however, a good indicator of ICU admission. Assessment of ICU indicators was again challenging due to the relatively limited cases that were not admitted to the ICU (5/23). Levels of PDGF-bb were elevated in those who were admitted to the ICU (Figure 6C). GM-CSF and VEGF were only detectable in those who were admitted to the ICU, though not in all individuals, and as such we were unable to perform a logistic regression due to perfect separation (Figure 6C). Hypoxemia induced changes in many cytokines, with 27 cytokines linearly correlated with SpO_2_ levels. After adjusting for age and sex, 15 cytokines differed between those with classified hypoxemia and those without (Figure 6D). Seven of these cytokines overlapped between analyses, one of which was IL-26. Using this cytokine alone, we could accurately classify 78.3% of hypoxemic events (Supplementary Figure S4). This accuracy increased to 82.6% when including G-CSF and IFNβ levels (Figure 6E).

### Cytokines associated with influenza severity

**Figure 7:**
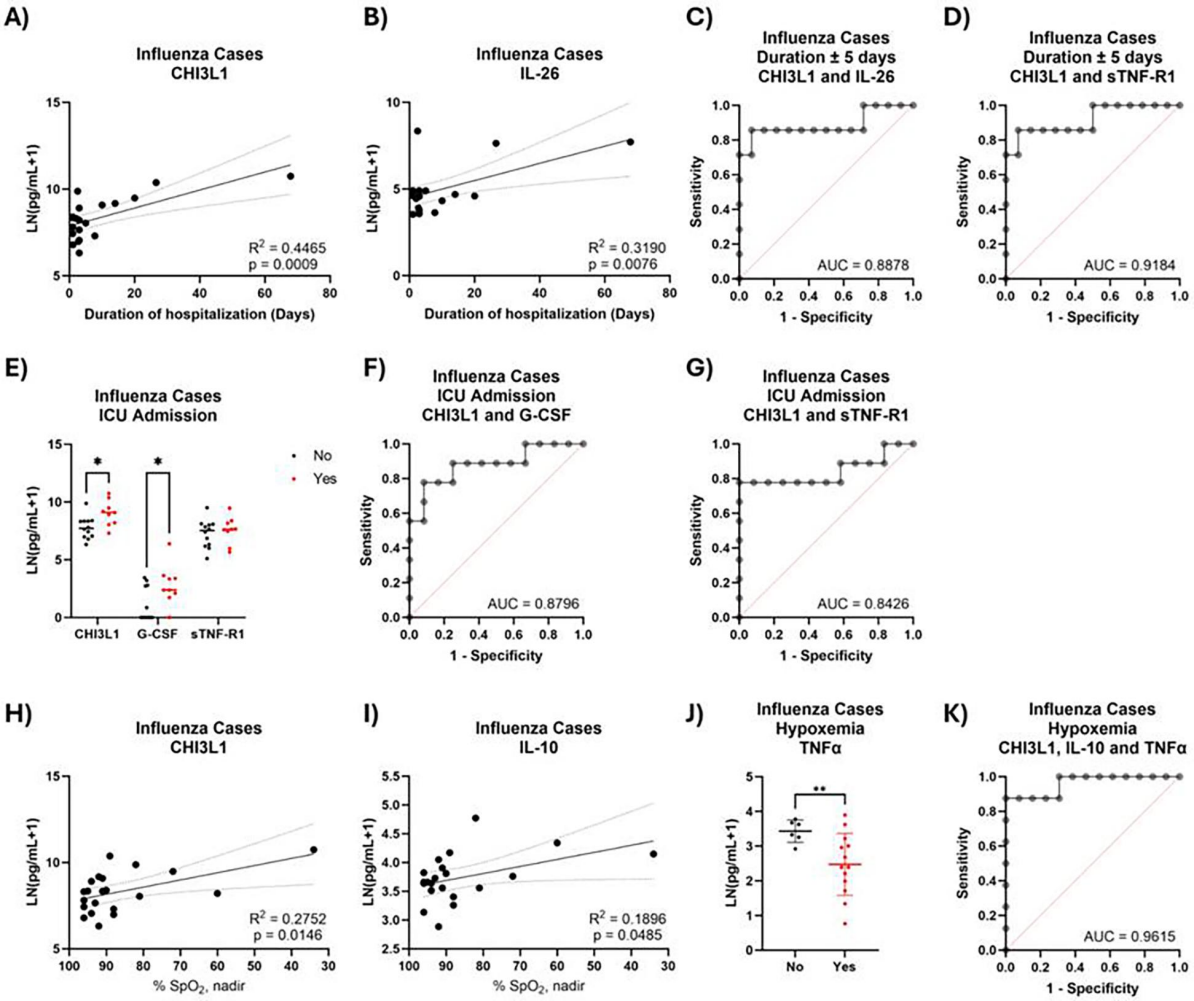
Plasma cytokine predictors of influenza severity. Duration of hospitalization in influenza^+^ cases against **(A)** CHI3L1 and **(B)** IL-26 levels. ROC curve generated by logistic regression for ±5-day hospitalization against **(C)** CHI3L1, IL-26 and CHI3L1:IL-26 or **(D)** CHI3L1, sTNF-R1, and CHI3L1:sTNF-R1 levels. **(E)** Levels of plasma cytokine in influenza^+^ cases admitted to the ICU or not. ROC curve generated by logistic regression for ICU admission against **(F)** CHI3L1, G-CSF and CHI3L1:G-CSF or **(G)** CHI3L1, sTNF-R1 and CHI3L1:sTNF-R1 levels. Plasma cytokine levels of **(H)** CHI3L1 or **(I)** IL-10 against % SpO_2_ levels or **(J)** TNFα levels against hypoxemic status. **(K)** ROC curve generated by logistic regression classifying hypoxemic status using CHI3L1, IL-10, TNFα, IL-10:CHI3L1, and IL-10:TNFα (*n* = 21).

Of those individuals with confirmed influenza infection (*N* = 26), we observed 12 cytokines which correlated with duration of hospitalization with an *R*^2^ > 0.2 (Supplementary Figure S5). All remained significant after accounting for age and sex. Using levels of CHI3L1 and IL-26 (Figures 7A,B), we could accurately classify longer- vs. shorter-term hospitalization 85.7% of the time (Figure 7C). One cytokine which had no correlation with duration, sTNF-R1, enhanced predictive power to 90.5% when coupled with CHI3L1 (Figure 7D). CHI3L1 levels were also elevated in those admitted to the ICU, as was G-CSF (Figure 7E). When coupled together, CHI3L1 and G-CSF correctly classified 81% of ICU admissions (Figure 7F). CHI3L1 and sTNF-R1 outperformed this model, with 85.7% of ICU admissions correctly classified (Figure 7G). For the final metric of disease severity, hypoxemia, we found that eight cytokines that were linearly correlated with SpO_2_ levels (Figures 7H,I and Supplementary Figure S6), six of which overlapped with those that correlated with duration. All remained significant after accounting for age and sex. Another cytokine, TNFα, was lower in hypoxemic cases and was included in downstream analyses (Figure 7J). We could accurately predict hypoxemia in 90.5% of cases using levels of CHI3L1, IL-10, and TNFα (Figure 7K).

### Cytokines associated with severity in CAP

**Figure 8:**
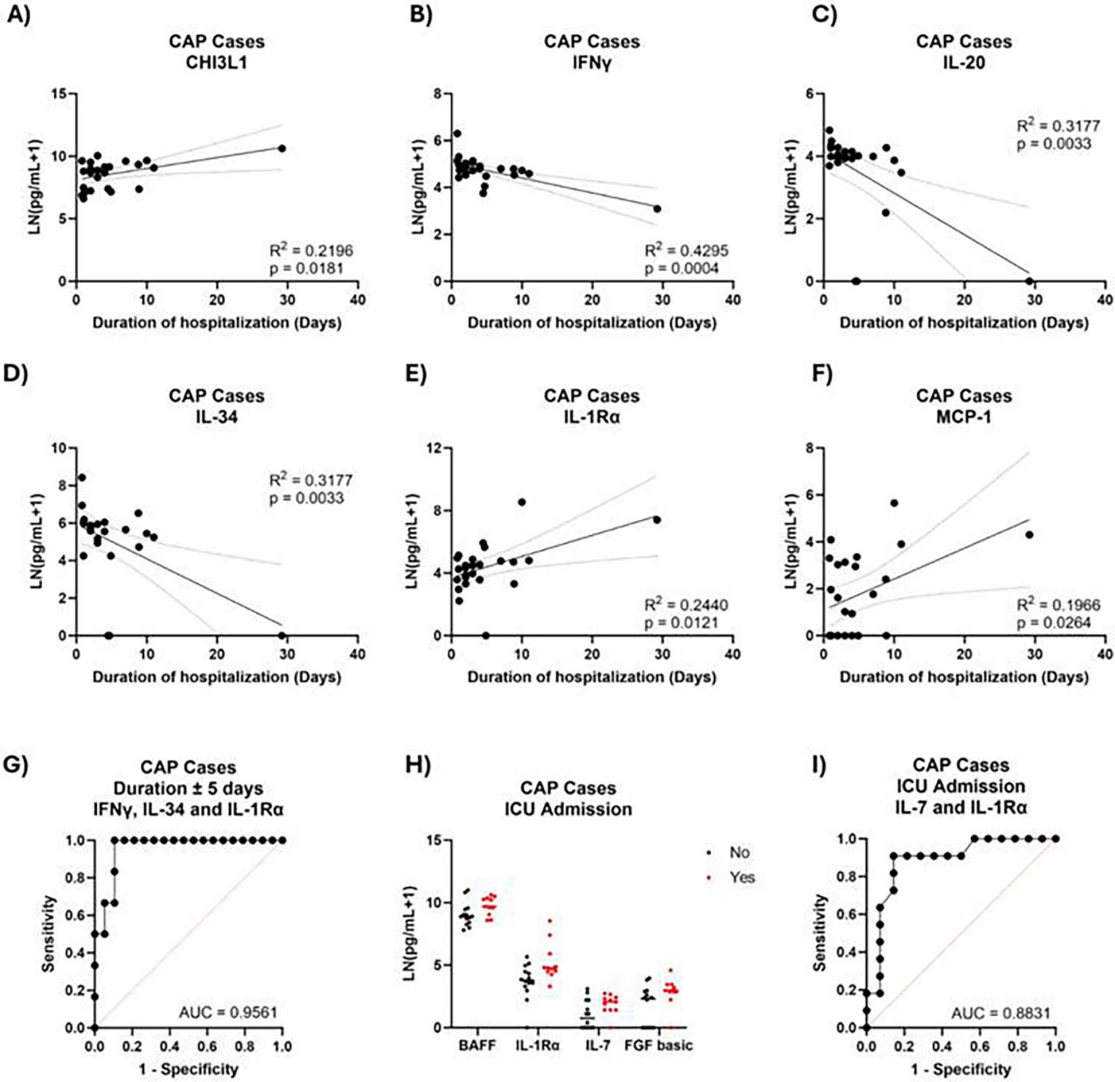
Plasma cytokine predictors of CAP severity. Duration of hospitalization in CAP cases against **(A)** CHI3L1, **(B)** IFNγ, **(C)** IL-20, **(D)** IL-34, **(E)** IL-1Rα, and **(F)** MCP-1. **(G)** ROC curve generated by logistic regression classifying ±5-day hospitalization in CAP cases using IFNγ, IL-34, and IL-1Rα. **(H)** Cytokine levels between CAP cases admitted to the ICU or not. **(I)** ROC curve generated by logistic regression classifying CAP ICU admission based on IL-7, IL-1Rα, and IL-7:IL-1Rα levels.

CAP infections broadly covered both confirmed and suspected bacterial pneumonias, as indicated in the clinical chart data of the participants. In CAP cases, six cytokines correlated with duration of hospitalization, but not age or sex (Figures 8A–F). Many of these cytokines also correlated with duration in other infections. Using levels of IFNγ, IL-34, and IL-1Rα, we accurately predicted longer- vs. shorter-term stays in 88% of cases (Figure 8G). In those admitted to the ICU, levels of BAFF, IL-7, FGF basic, and IL-1Rα tended to be higher and did not correlate with age or sex (Figure 8H). Using IL-7 and IL-1Rα, we correctly classified 84% of ICU admissions (Figure 8I). Hypoxemia was uncommon in our cohort of CAP cases (5/25). No cytokine linearly correlated with SpO_2_ levels or with hypoxemic status after controlling for age and sex.

## Discussion

Among children hospitalized for pneumonia-related illnesses over a 10-year period at the University of Pittsburgh Medical Center Children's Hospital, the most frequent infections were rhinovirus/enterovirus (24.4%), RSV (21.8%), and influenza virus (16.7%). Of the 156 participants enrolled, 26 (16.7%) had a confirmed coinfection (viral–viral, viral–bacterial, or bacterial–bacterial). These findings are consistent with other studies (15, 16). While we did not observe any cytokines in our panels that were strongly correlated with severity across all pneumonias, we did find strong associations within specific infections.

Consistent with other reports, duration of hospitalization with RSV infection correlated with T helper 2 (Th2) response cytokines, including IL-4, IL-5, and IL-13 (17, 18). We also observed a strong association between MIP-1α levels and longer hospitalization, confirming previous studies on RSV severity (19). Hypoxemia among RSV-positive individuals could also be categorized based on IL-26, G-CSF, and IFNβ levels. In contrast with another study, we found decreased IL-10 levels in the plasma of hypoxemic RSV-positive individuals (20).

For individuals hospitalized with influenza virus infection, plasma CHI3L1 levels were highly indicative of severe disease as indicated in our study (≥5 day hospitalization, ICU admission, hypoxemic). Increased CHI3L1 was a major component of all metrics of severity. CHI3L1 levels have previously been associated with age (21, 22). Interestingly, another study from our laboratory assessed plasma cytokine levels of inpatient and outpatient adults with influenza infection. There was no association of CHI3L1 with severity in adults; however, levels of CHI3L1 in adults were similar to those observed in this pediatric cohort with extremely prolonged hospitalization (10^+^ days) (23). Future investigations into the strength of CHI3L1 as a predictor for influenza severity should continue to account for age as a factor.

Although hRV/EV cases accounted for most infections within our cohort, we were unable to find strong associations of any cytokine(s) with severity. This could be in part due to the association of duration of hospitalization with age of the participants. We had similar issues separating severity using ICU admission, as most hRV/EV-positive individuals required intensive care. MMP-3 and pentraxin-3 emerged as potential candidates for identifying severe cases but require a larger cohort for more stringent verification. Though IL-2 and MMP-3 correlated with SpO_2_ levels, they were only moderately successful in classifying hypoxemic status. Another confounding factor in assessing severity could be in the identification of the hRV/EV pathogen itself. Rhinovirus families separate into type A, B, and C, with type C being associated with more severe disease (24). It is possible that separating rhinovirus from pneumonia-inducing enterovirus infections, or distinguishing between rhinovirus strains, may yield clearer associations in the future.

Although our CAP cohort was a catch-all basin for all non-viral pneumonias, we nevertheless identified strong associations with a subset of cytokines and severity. IL-1Rα was present in both logistic regression models categorizing duration and ICU admission. IL-1Rα levels have previously been reported to be elevated in acute phase response to infection and may be indicative of *Streptococcus pneumoniae* infection (25, 26). In our cohort, IL-1Rα levels of *S. pneumoniae*-positive cultures were not higher than overall CAP levels; however, not all CAP cases had a positively identified pathogen, so additional *S. pneumoniae*-positive infections could be present in the CAP category (data not shown).

Perhaps most important among our results is the discovery of a potential grouping of three cytokines (IL-6, MMP-2, and LIGHT) that may be able to differentiate viral and non-viral CAP infections. Notably, the positive predictive value for viral infection was much greater than for CAP, indicating the ability to identify viral infection more effectively than CAP. Within our cohort, we only tested for the presence of common respiratory pathogens, including influenza virus, RSV, rhinovirus/enterovirus, human metapneumovirus, adenovirus, and parainfluenza viruses 1–4. This is by no means an exhaustive panel of all respiratory viruses that could cause pneumonia, and as such, there is a possibility that a viral-induced pneumonia may have been categorized as CAP. In addition, this study did not specifically require cultures from nasal swabs, sputum, or bronchoalveolar lavage, which limited our ability to identify bacterial species within our cohort, unless specifically requested by the participant's physician. Current CAP biomarkers, such as procalcitonin and CRP, have demonstrated similar limitations as our cytokine approach. It is intriguing to consider combining these markers with inflammatory cytokines. As such, the cytokines identified herein still require validation with an additional cohort to confirm their viability as pneumonia etiology markers. Nevertheless, they provide promising potential to aid in the separation of viral and non-viral infection, thereby assisting healthcare providers in the administration of antibiotic courses.

Of those participants with confirmed viral–bacterial coinfections (*N* = 9), we had collected samples from seven. These seven samples, when classified as being of viral or bacterial origin, skewed heavily toward viral prediction, suggesting that viral infection, rather than bacterial infection, drives more of the cytokine profile during coinfection. These patients are very interesting clinically and often present with severe infection. While animal models have been used to study immunopathogenesis, few studies have focused specifically on this subgroup. Further studies designed to differentiate cytokine profiles between respiratory viral and bacterial pneumonia will be needed to verify our results in this small subset of participants.

We also attempted to differentiate the distinct viral infections identified from one another. Cytokine levels did vary among the different confirmed viral infections; however, given the relatively small number of positive cases for each infection and the lack of a validation cohort, it is challenging to determine the strength of these cytokine combinations. An additional confounding factor is the significant age differences between participants with RSV infection, hRV/EV infection, and the rest of the cohort. Strengths of the study included the collection of samples across multiple respiratory infection seasons. Findings from this study likely highlight conserved cytokine responses to infection that are not dependent on the predominant circulating strains for each season. Limitations of the study include the relatively limited cytokine concentration differences observed. It is unlikely that a single cytokine would have predictive value in this setting and thus our approach focused on combinations of markers to increase predictive power. Another key limitation is the overall sample size and lack of a validation cohort. Although we incorporated validation within the cohort into our machine learning analysis to support viral vs. CAP detection and confirmed linear regression findings, these caveats highlight the need for larger, longitudinally robust, multicenter studies to validate our results and define the clinical potential of the findings.

## Data Availability

The original contributions presented in the study are included in the article/Supplementary Material, further inquiries can be directed to the corresponding author.

## References

[1] Gruber C Keil T Kulig M Roll S Wahn U Wahn V History of respiratory infections in the first 12 yr among children from a birth cohort. Pediatr Allergy Immunol. (2008) 19(6):505–12. 10.1111/j.1399-3038.2007.00688.x 18167154

[2] GBD 2019 Diseases and Injuries Collaborators. Global burden of 369 diseases and injuries in 204 countries and territories, 1990–2019: a systematic analysis for the Global Burden of Disease Study 2019. Lancet. (2020) 396(10258):1204–22. 10.1016/S0140-6736(20)30925-9 33069326 PMC7567026

[3] Jain S Williams DJ Arnold SR Ampofo K Bramley AM Reed C Community-acquired pneumonia requiring hospitalization among U.S. children. N Engl J Med. (2015) 372(9):835–45. 10.1056/NEJMoa1405870 25714161 PMC4697461

[4] Pratt MTG Abdalla T Richmond PC Moore HC Snelling TL Blyth CC Prevalence of respiratory viruses in community-acquired pneumonia in children: a systematic review and meta-analysis. Lancet Child Adolesc Health. (2022) 6(8):555–70. 10.1016/S2352-4642(22)00092-X 35636455

[5] Esposito S Daleno C Prunotto G Scala A Tagliabue C Borzani I Impact of viral infections in children with community-acquired pneumonia: results of a study of 17 respiratory viruses. Influenza Other Respir Viruses. (2013) 7(1):18–26. 10.1111/j.1750-2659.2012.00340.x 22329841 PMC5780730

[6] Perez A Lively JY Curns A Weinberg GA Halasa NB Staat MA Respiratory virus surveillance among children with acute respiratory illnesses—new vaccine surveillance network, United States, 2016–2021. MMWR Morb Mortal Wkly Rep. (2022) 71(40):1253–9. 10.15585/mmwr.mm7140a1 36201373 PMC9541034

[7] Hausfater P. Biomarkers and infection in the emergency unit. Med Mal Infect. (2014) 44(4):139–45. 10.1016/j.medmal.2014.01.002 24556451

[8] Povoa P. C-reactive protein: a valuable marker of sepsis. Intensive Care Med. (2002) 28(3):235–43. 10.1007/s00134-002-1209-6 11904651

[9] Assicot M Gendrel D Carsin H Raymond J Guilbaud J Bohuon C. High serum procalcitonin concentrations in patients with sepsis and infection. Lancet. (1993) 341(8844):515–8. 10.1016/0140-6736(93)90277-N 8094770 PMC7141580

[10] Erixon ER Cunningham KJ Schlicher AN Dajud MV Ferguson AM Fondell AW Use of procalcitonin for identification of cobacterial pneumonia in pediatric patients. J Pediatr Pharmacol Ther. (2020) 25(5):445–50. 10.5863/1551-6776-25.5.445 32641915 PMC7337135

[11] Rodriguez AH Aviles-Jurado FX Diaz E Schuetz P Trefler SI Sole-Violan J Procalcitonin (PCT) levels for ruling-out bacterial coinfection in ICU patients with influenza: a CHAID decision-tree analysis. J Infect. (2016) 72(2):143–51. 10.1016/j.jinf.2015.11.007 26702737

[12] Declercq J De Leeuw E Lambrecht BN. Inflammasomes and IL-1 family cytokines in SARS-CoV-2 infection: from prognostic marker to therapeutic agent. Cytokine. (2022) 157:155934. 10.1016/j.cyto.2022.155934 35709568 PMC9170572

[13] Mayne ES George JA Louw S. Assessing biomarkers in viral infection. Adv Exp Med Biol. (2023) 1412:159–73. 10.1007/978-3-031-28012-2_8 37378766

[14] Shojaee A Rafiee R Hosseinzadeh M Saboori M. Prognostic value of interleukin-6 serum levels in hospitalized COVID-19 patients: a case-control study in Iran. Health Sci Rep. (2024) 7(7):e2232. 10.1002/hsr2.2232 38978767 PMC11228099

[15] Bahceci I Yildiz IE Duran OF Soztanaci US Kirdi Harbawi Z Senol FF Secondary bacterial infection rates among patients with COVID-19. Cureus. (2022) 14(2):e22363. 10.7759/cureus.22363 35371794 PMC8938257

[16] Klein EY Monteforte B Gupta A Jiang W May L Hsieh YH The frequency of influenza and bacterial coinfection: a systematic review and meta-analysis. Influenza Other Respir Viruses. (2016) 10(5):394–403. 10.1111/irv.12398 27232677 PMC4947938

[17] Pinto RA Arredondo SM Bono MR Gaggero AA Diaz PV. T helper 1/T helper 2 cytokine imbalance in respiratory syncytial virus infection is associated with increased endogenous plasma cortisol. Pediatrics. (2006) 117(5):e878–86. 10.1542/peds.2005-2119 16618789

[18] Vu LD Siefker D Jones TL You D Taylor R DeVincenzo J Elevated levels of type 2 respiratory innate lymphoid cells in human infants with severe respiratory syncytial virus bronchiolitis. Am J Respir Crit Care Med. (2019) 200(11):1414–23. 10.1164/rccm.201812-2366OC 31237777 PMC6884055

[19] Garofalo RP Patti J Hintz KA Hill V Ogra PL Welliver RC. Macrophage inflammatory protein-1alpha (not T helper type 2 cytokines) is associated with severe forms of respiratory syncytial virus bronchiolitis. J Infect Dis. (2001) 184(4):393–9. 10.1086/322788 11471095

[20] Alonso Fernandez J Roine I Vasquez A Caneo M. Soluble interleukin-2 receptor (sCD25) and interleukin-10 plasma concentrations are associated with severity of primary respiratory syncytial virus (RSV) infection. Eur Cytokine Netw. (2005) 16(1):81–90. 15809211

[21] Cetin M Kocaman SA Canga A Kirbas A Yilmaz A Erdogan T Elevated serum YKL-40 level predicts myocardial reperfusion and in-hospital MACE in patients with STEMI. Herz. (2013) 38(2):202–9. 10.1007/s00059-012-3671-4 22955689

[22] Kamle S Ma B He CH Akosman B Zhou Y Lee CM Chitinase 3-like-1 is a therapeutic target that mediates the effects of aging in COVID-19. JCI insight. (2021) 6(21):e148749. 10.1172/jci.insight.148749 34747367 PMC8663553

[23] Luciani LL Miller LM Zhai B Clarke K Hughes Kramer K Schratz LJ Blood inflammatory biomarkers differentiate inpatient and outpatient coronavirus disease 2019 from influenza. Open Forum Infect Dis. (2023) 10(3):ofad095. 10.1093/ofid/ofad095 36949873 PMC10026548

[24] Sayama A Okamoto M Tamaki R Saito-Obata M Saito M Kamigaki T Comparison of rhinovirus A-, B-, and C-associated respiratory tract illness severity based on the 5′-untranslated region among children younger than 5 years. Open Forum Infect Dis. (2022) 9(10):ofac387. 10.1093/ofid/ofac387 36267245 PMC9579461

[25] Endeman H Meijvis SC Rijkers GT van Velzen-Blad H van Moorsel CH Grutters JC Systemic cytokine response in patients with community-acquired pneumonia. Eur Respir J. (2011) 37(6):1431–8. 10.1183/09031936.00074410 20884746

[26] Verma N Awasthi S Pandey AK Gupta P. Assessment of interleukin 1 receptor antagonist (IL-1RA) levels in children with and without community acquired pneumonia: a hospital based case-control study. J Trop Pediatr. (2023) 69(6):fmad040. 10.1093/tropej/fmad040 37994793

