# Supplementary material for: Predicting infectious etiology and severity in hospitalized pediatric pneumonia using blood cytokine biomarkers
Source: Front Pediatr. 2025 Dec 19;13:1693879. doi: 10.3389/fped.2025.1693879 (PMC12757334; doi:10.3389/fped.2025.1693879)
Supplement: Supplementary file 1 [file Datasheet1.pdf]

Supplemental Table 1: Two-Way ANOVA *p* values of Infections vs. Cytokines

	sCD30	IL-22	IL-26	IL-27p28	IFNλ2	IL-32	IL-34	LIGHT (TNFSF14)	OCN	OPN
hRV/EV vs. CAP	0.8869	0.0315	0.955	0.2149	0.1847	0.018	0.3129	0.0002	0.6293	0.28
RSV vs. CAP	0.1628	0.3573	0.054	0.2082	0.9959	0.997	<0.0001	0.9975	0.2791	0.2192
RSV vs. hRV/EV	0.0257	0.8762	0.013	0.0008	0.1249	0.014	0.0094	0.0028	0.0147	0.0014
hMPV vs. CAP	>0.9999	0.2793	0.998	0.9935	0.9497	0.957	0.1139	0.0721	0.9958	>0.9999
hMPV vs. hRV/EV	0.9452	>0.9999	0.943	0.8596	0.198	0.652	0.8234	0.998	0.9804	0.7118
hMPV vs. RSV	0.6496	0.9616	0.551	0.3596	0.9914	0.9	0.7072	0.1543	0.4482	0.6013
Influenza vs. CAP	>0.9999	0.9566	0.999	0.2659	0.8347	0.979	0.9991	<0.0001	>0.9999	>0.9999
Influenza vs. hRV/EV	0.9523	0.2611	0.994	>0.9999	0.0231	0.143	0.5472	0.9618	0.7387	0.3244
Influenza vs. RSV	0.1761	0.8352	0.049	0.0013	0.9719	0.92	<0.0001	0.0002	0.3229	0.3311
Influenza vs. hMPV	0.9994	0.6084	0.991	0.8846	>0.9999	0.999	0.2015	0.9344	0.9979	>0.9999

Pentraxin-3	sTNF-R1	sTNF-R2	IL-1Rα	IL-6	FGF basic	G-CSF	GM-CSF	IP-10	MCP-1	PDGF-bb
0.587	0.2972	0.0174	0.016	<0.0001	0.0244	0.997	0.044	0.022	0.583	0.1259
0.0408	0.6436	0.9522	0.485	0.0223	>0.9999	0.999	0.3755	0.994	0.037	0.0217
0.705	0.018	0.004	1E-04	0.4755	0.05	0.98	0.9063	0.013	6E-04	0.9669
0.9751	0.9996	0.9999	0.471	0.9732	0.8759	0.002	0.7495	0.404	0.012	0.2708
0.9953	0.8147	0.3231	0.003	0.0015	0.8337	0.001	0.9627	0.002	4E-04	0.9962
0.6953	0.7991	0.9701	0.988	0.0713	0.8951	0.006	>0.9999	0.627	0.756	>0.9999
0.9683	0.8842	>0.9999	0.982	0.1611	0.8654	0.967	0.9986	0.88	0.999	0.1988
0.9453	0.8922	0.0466	0.007	0.1321	0.3574	0.891	0.1475	0.002	0.497	0.9998
0.2677	0.2061	0.9435	0.86	0.9571	0.9013	0.997	0.6292	0.989	0.121	0.9308
0.9999	0.9931	>0.9999	0.745	0.2237	0.9989	0.013	0.8701	0.832	0.03	0.9894
