# Supplementary material for: Predicting infectious etiology and severity in hospitalized pediatric pneumonia using blood cytokine biomarkers
Source: Front Pediatr. 2025 Dec 19;13:1693879. doi: 10.3389/fped.2025.1693879 (PMC12757334; doi:10.3389/fped.2025.1693879)
Supplement: Supplementary file 2 [file Datasheet2.pdf]

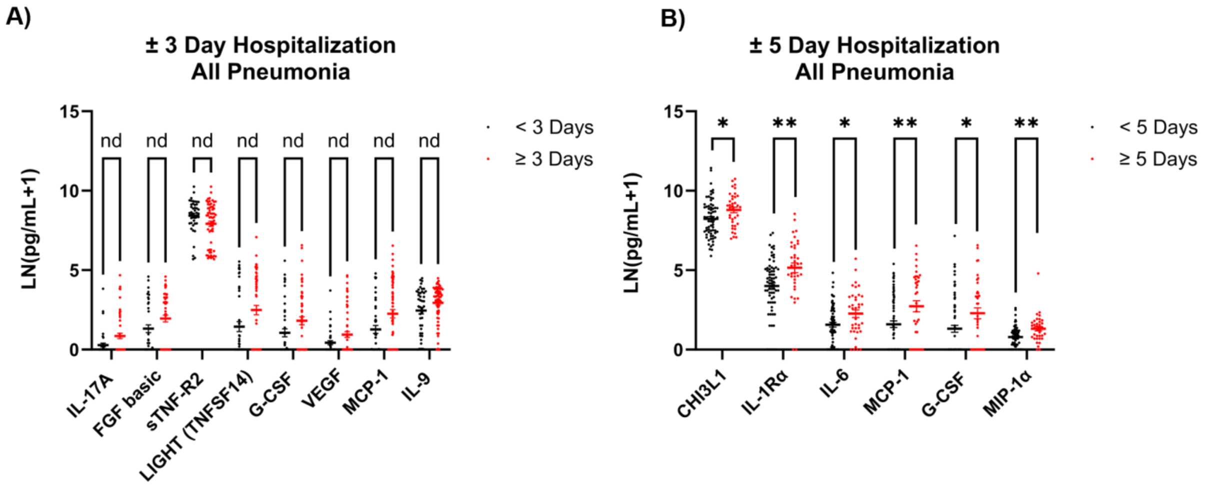

Supplemental Figure 1: Plasma Cytokine Levels in All Pneumonia Patients.

Plasma cytokine levels for participants separated by A) ±3-day or B) ±5-day hospitalization. Cytokines listed were significant or trended towards significance for their identified comparison (n=107). *q<0.05 **q<0.01

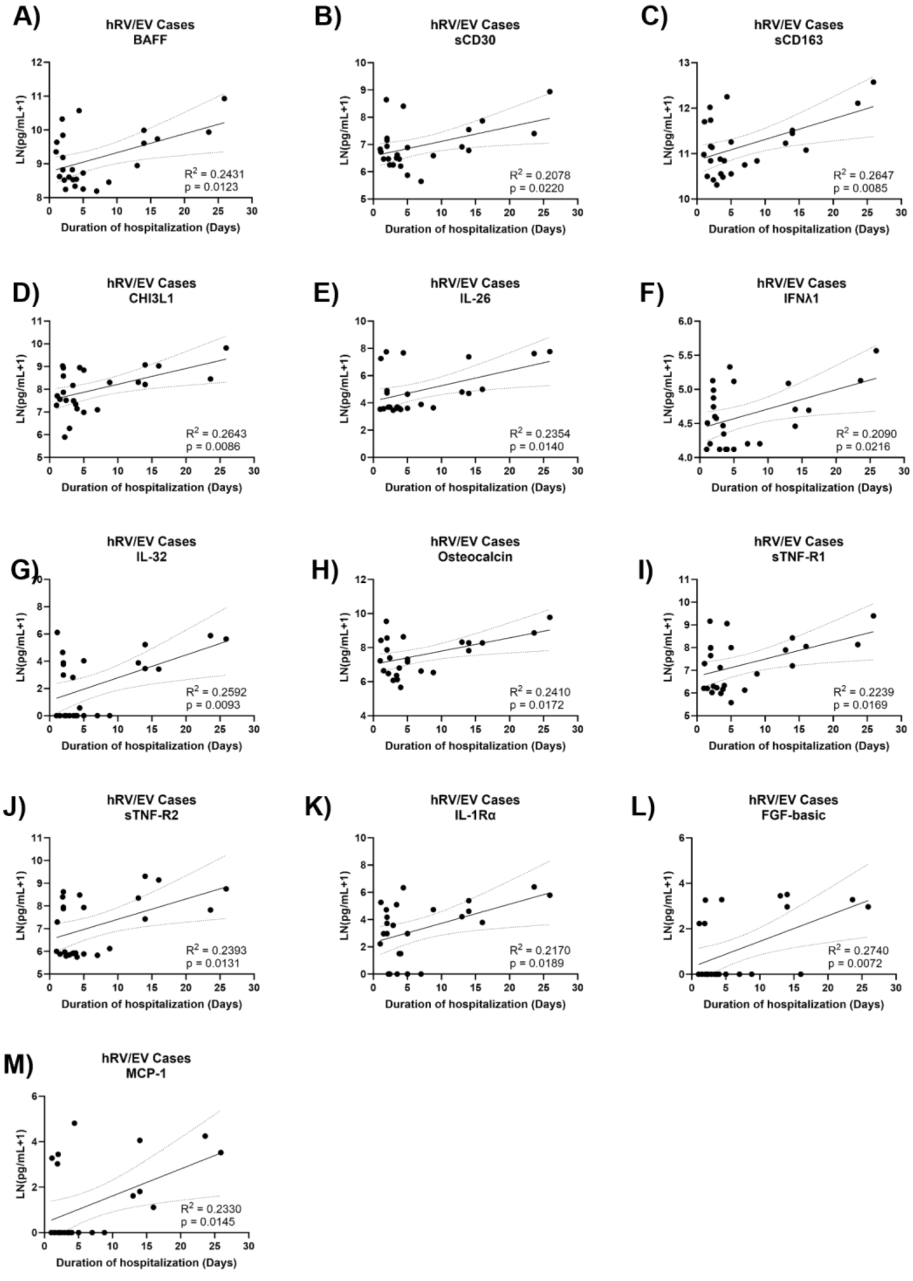

Supplemental Figure 2: Plasma Cytokine Levels versus Hospitalization Duration in hRV/EV Cases.

Linear regressions for all cytokines which significantly correlated with duration of hospitalization in hRV/EV$^+$ cases. A) BAFF, B) sCD30, C) sCD163, D) CHI3L1, E) IL-26, F) IFNλ1, G) IL-32, H) Osteocalcin (OCN), I) sTNF-R1, J) sTNF-R2, K) IL-1Rα, L) FGF-basic and M) MCP-1 (n=25).

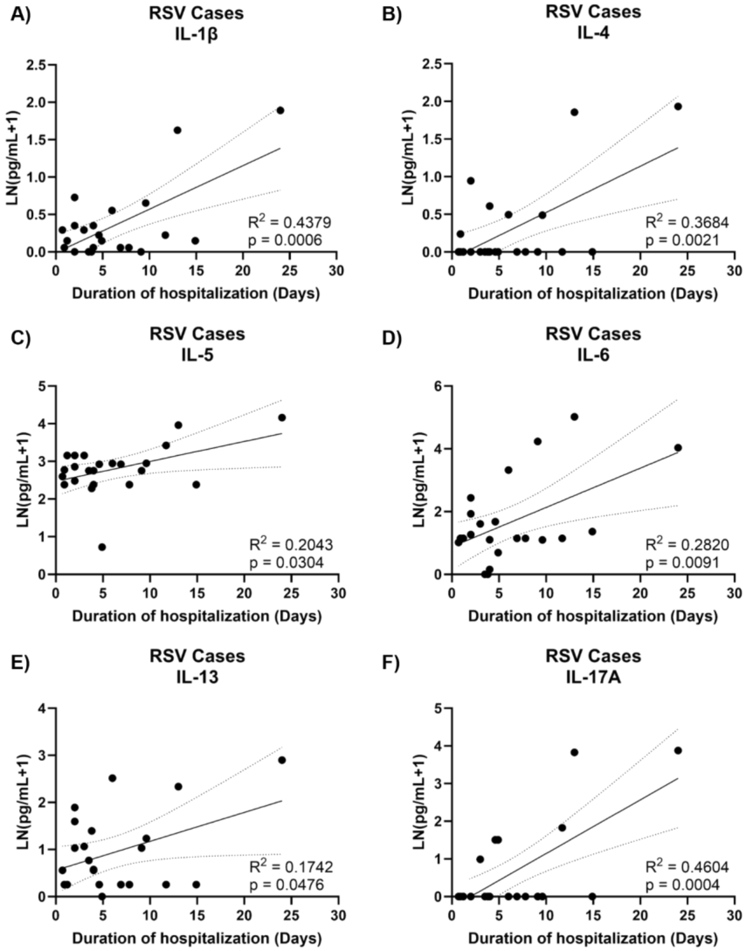

Supplemental Figure 3: Plasma Cytokines versus Hospitalization Duration for RSV Cases

Linear regressions of plasma cytokine levels in confirmed RSV^+ cases against duration of hospitalization for A) IL-1β, B) IL-4, C) IL-5, D) IL-6, E) IL-13 and F) IL-17A (n=23).

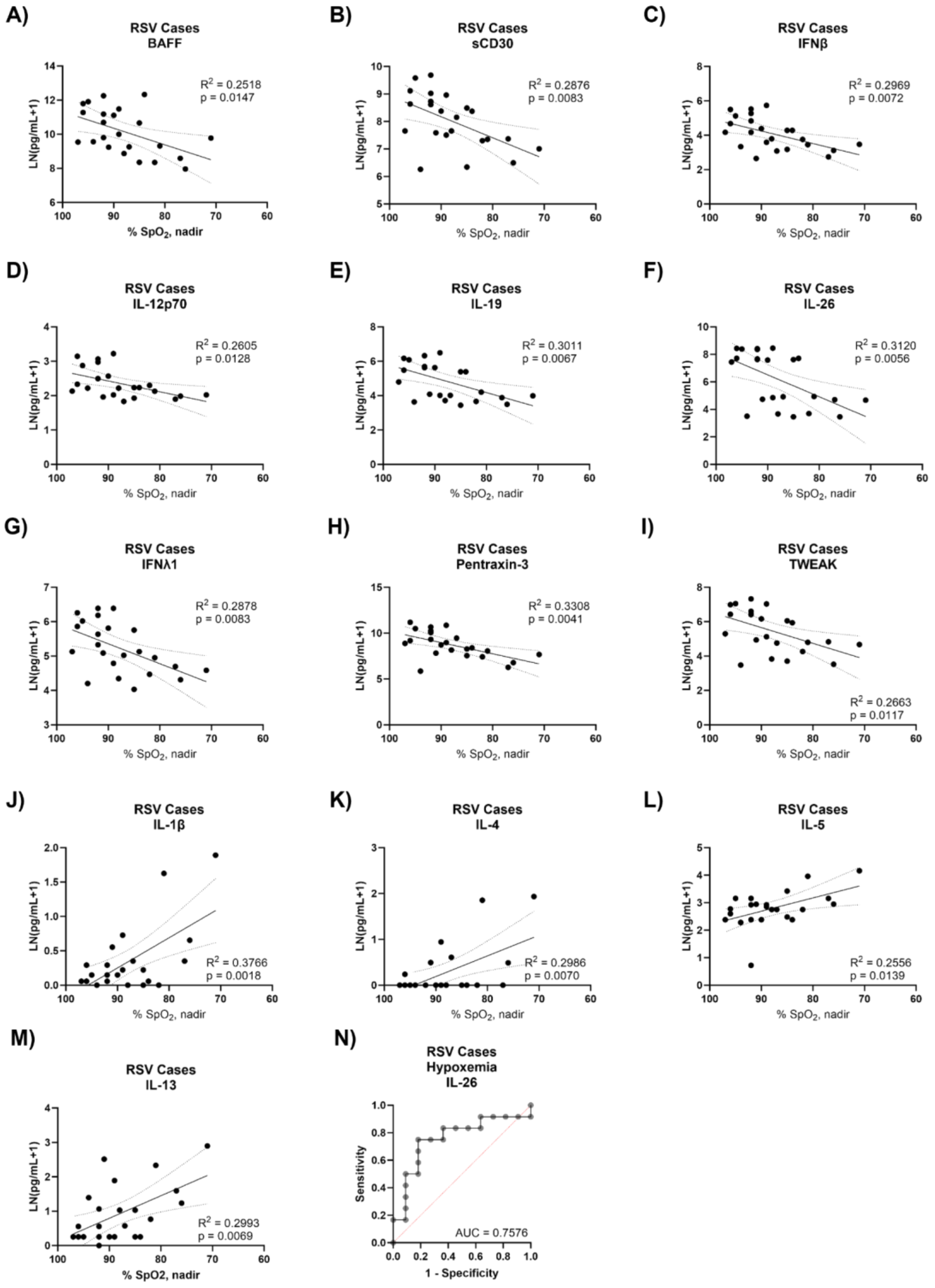

Supplemental Figure 4: Plasma Cytokines versus Hypoxemic Status in RSV Cases
Linear regression in RSV⁺ cases for % SpO_2 levels against A) BAFF, B) sCD30, C) IFNβ, D) IL-12p70, E) IL-19, F) IL-26, G) IFNλ1, H) Pentraxin-3, I) TWEAK, J) IL-1β, K) IL-4, L) IL-5, and M) IL-13 levels. N) ROC curve generated by logistic regression classifying hypoxemic status in RSV⁺ cases using IL-26 (n=23).

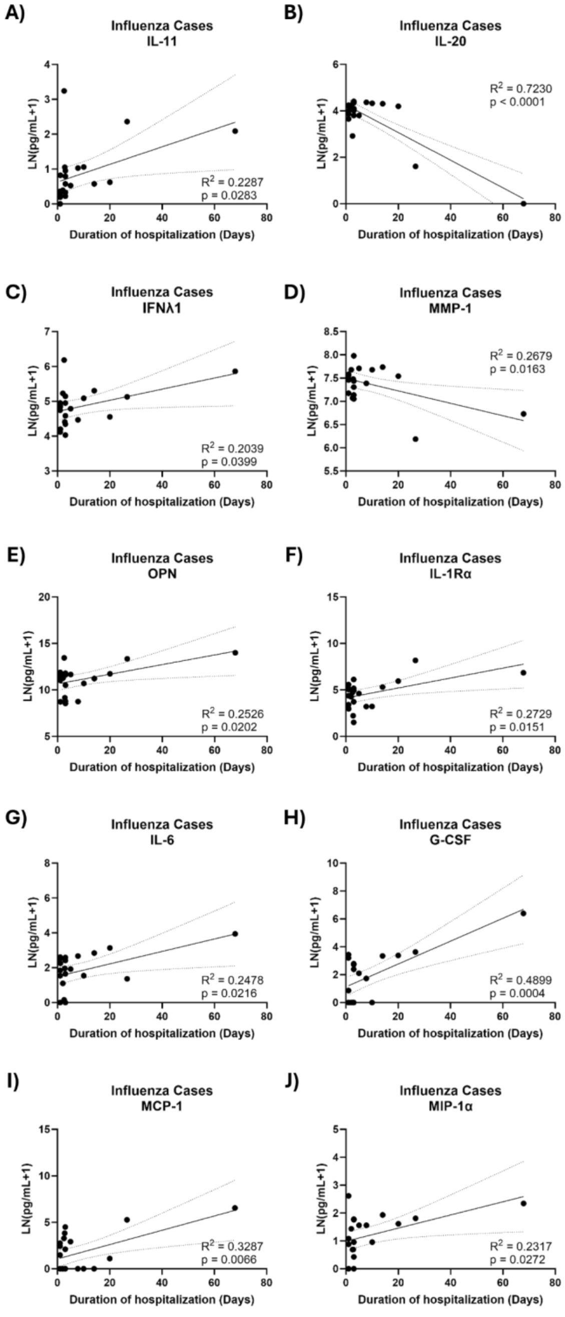

Supplemental Figure 5:Plasma Cytokines versus Hospitalization Duration for Influenza Cases.
In influenza^{+} cases, duration of hospitalization linearly correlated with plasma levels of A) IL-11, B) IL-20, C) IFNλ1, D) MMP-1, E) Osteopontin (OPN), F) IL-1rα, G) IL-6, H) G-CSF, I) MCP-1 and J) MIP-1α levels (n=21).

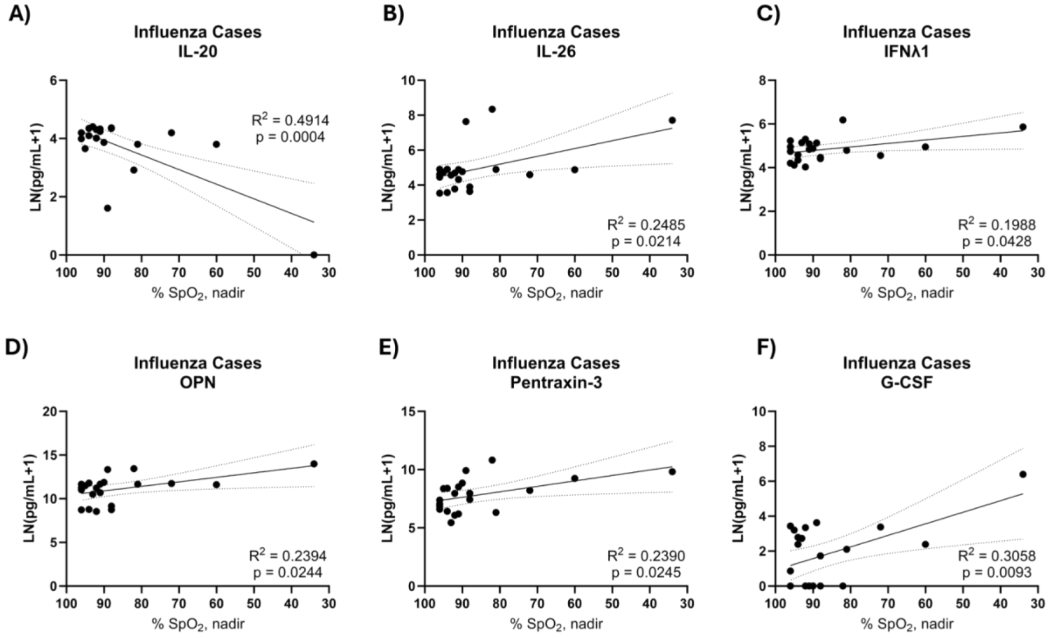

Supplemental Figure 6: Plasma Cytokines versus Hypoxemic Status in Influenza Cases.

Blood oxygenation levels in influenza$^+$ cases against levels of A) IL-20, B) IL-26, C) IFNλ1, D) Osteopontin (OPN), E) Pentraxin-3, and F) G-CSF (n=21).
